# Patterns of genetic variation across inversions: geographic variation in the *In*(*2L*)*t* inversion in populations of *Drosophila melanogaster* from eastern Australia

**DOI:** 10.1186/1471-2148-13-100

**Published:** 2013-05-20

**Authors:** W Jason Kennington, Ary A Hoffmann

**Affiliations:** 1 Centre for Evolutionary Biology, The University of Western Australia, Crawley, WA, 6009, Australia; 2 Departments of Zoology and Genetics, The University of Melbourne, Melbourne, Vic, 3010, Australia

**Keywords:** Inversions, Coadaptation, Genetic variation, Latitudinal cline, Population structure, Natural selection

## Abstract

**Background:**

Chromosomal inversions are increasingly being recognized as important in adaptive shifts and are expected to influence patterns of genetic variation, but few studies have examined genetic patterns in inversion polymorphisms across and within populations. Here, we examine genetic variation at 20 microsatellite loci and the alcohol dehydrogenase gene (*Adh*) located within and near the *In*(*2L*)*t* inversion of *Drosophila melanogaster* at three different sites along a latitudinal cline on the east coast of Australia.

**Results:**

We found significant genetic differentiation between the standard and inverted chromosomal arrangements at each site as well as significant, but smaller differences among sites in the same arrangement. Genetic differentiation between pairs of sites was higher for inverted chromosomes than standard chromosomes, while inverted chromosomes had lower levels of genetic variation even well away from inversion breakpoints. Bayesian clustering analysis provided evidence of genetic exchange between chromosomal arrangements at each site.

**Conclusions:**

The strong differentiation between arrangements and reduced variation in the inverted chromosomes are likely to reflect ongoing selection at multiple loci within the inverted region. They may also reflect lower effective population sizes of *In*(*2L*)*t* chromosomes and colonization of Australia, although there was no consistent evidence of a recent bottleneck and simulations suggest that differences between arrangements would not persist unless rates of gene exchange between them were low. Genetic patterns therefore support the notion of selection and linkage disequilibrium contributing to inversion polymorphisms, although more work is needed to determine whether there are spatially varying targets of selection within this inversion. They also support the idea that the allelic content within an inversion can vary between geographic locations.

## Background

Chromosome inversions occur when a chromosome breaks in two places and the segment between the breakpoints is re-inserted in the reverse orientation. In *Drosophila* and other Diptera, inverted and noninverted (standard) forms of chromosomes often coexist within the same population. These inversion polymorphisms can be identified by examining the banding patterns of chromosomes in the larval salivary gland cells, and by the formation of loops during chromosomal pairing between inverted and standard arrangements, making them convenient genetic markers for studying evolution [1,2]. Studies on inversion frequency changes in natural and laboratory populations of *Drosophila* by Dobzhansky and his colleagues provided early evidence that inversion polymorphisms are under strong selection and adaptation [3,4]. Since then evidence for selection and adaptation involving inversions have been found in increasing numbers of species including plants [5], seaweed flies [6], butterflies [7], *Anopheles* mosquitoes [8], fruit flies [9] and humans [10]. Inversions are also thought to play a role in the evolution of sex chromosomes and speciation [1].

The spread and maintenance of inversion polymorphisms is thought to be due to their impact on linkage disequilibrium. Inversions maintain associations between alleles because crossing over between inverted and standard arrangements gives rise to nonfunctional meiotic products. Under the coadaptation hypothesis proposed by Dobzhansky, inversions have selective value because they hold together favourable combinations of alleles [3,11]. A crucial aspect of the hypothesis is that alleles at loci within the inversion have epistatic interactions that increase fitness. Heterosis and the idea that the allelic content of the inversion evolves after inversions arise are also assumed, leading to different alleles in populations within the same inversion [12,13].

An alternative hypothesis is that inversions have selective value because they bring together two or more alleles that are adapted to local conditions [14]. With this model, no epistasis is needed for the inversion to gain a fitness advantage, so the mechanism can operate even when alleles are adapting to different environmental variables. It also does not require sets of alleles to become coadapted within a chromosomal arrangement in a population, or for the presence of heterosis. This mechanism may therefore occur much more frequently than mechanisms involving coadaptation [2]. An inversion harbouring locally adapted alleles will go to fixation unless a polymorphism is maintained by migration or balancing selection. Other explanations why inversions spread through populations include direct selection on the inversion (rather than its effects on recombination) arising from a mutation at the breakpoints, underdominance and overdominance [1,2].

Much of the empirical support for the idea that inversions are locally adapted comes from laboratory experiments on *Drosophila* reviewed in [11]. Several studies have shown that changes in inversion frequencies in population cages depend on when and where samples from natural populations were taken [15] and how they have been maintained [16,17]. More recently, Lowry and Willis [5] have used reciprocal transplant experiments involving outbred lines where inversion chromosomal arrangements were introgressed into different genetic backgrounds to demonstrate local adaptation in the yellow monkey flower.

Molecular studies provide further evidence that inversions evolve over time and are involved in local adaptation. Levels of linkage disequilibrium (LD) and nucleotide divergence between inverted and standard chromosome arrangement change over time, and become reduced towards the middle of the inversion where multiple crossover and gene conversion are expected to be higher see [1], although this is not always the case (e.g., [18]). Patterns of LD within inversions may also reflect selection as well as recombination and historical processes; in *Drosophila pseudoobscura* and *Drosophila melanogaster*, LD between genes within inversion decreases as they are situated further apart, but some nonadjacent genes maintain high LD with regions of low LD between them, suggesting selection at loci across the inverted region [13,19].

By contrast, molecular evidence for local adaptation is surprisingly scarce and inconsistent. Allozyme studies on *D*. *pseudoobscura* have shown that the same inversions from different populations have unique combinations of alleles [20,21]. However, samples sizes in these studies tended to be small [13] and nucleotide sequences of genes situated within inversions show no significant differences among populations within the same arrangements [13,22]. In mosquitoes, patterns of nucleotide divergence between chromosomal arrangements are inconsistent with neutral models [23], but these patterns are not always found [24] and clear footprints of selective sweeps or balancing selection on genes within inversions are uncommon [23,24]. However, signatures of selection have been reported in genes within inversions in *Drosophila* (e.g., [18,25]).

Here, we examine geographic variation in the *In*(*2L*)*t* inversion in populations of *D*. *melanogaster* along the east coast of Australia. This chromosome arrangement is located in the middle of the left arm on chromosome 2 (breakpoints 22D3-E1 and 34A8-9) and has been the focus of many studies due its close proximity to the *alcohol dehydrogenase* (*Adh*) locus. As found on other continents, both *Adh* and *In*(*2L*)*t* show latitudinal clines in eastern Australia, with higher frequencies of the *Adh*^*S*^ allele and *In*(*2L*)*t* at lower latitudes, providing strong evidence that natural selection is maintaining the polymorphisms [26,27].

**Figure 1:**
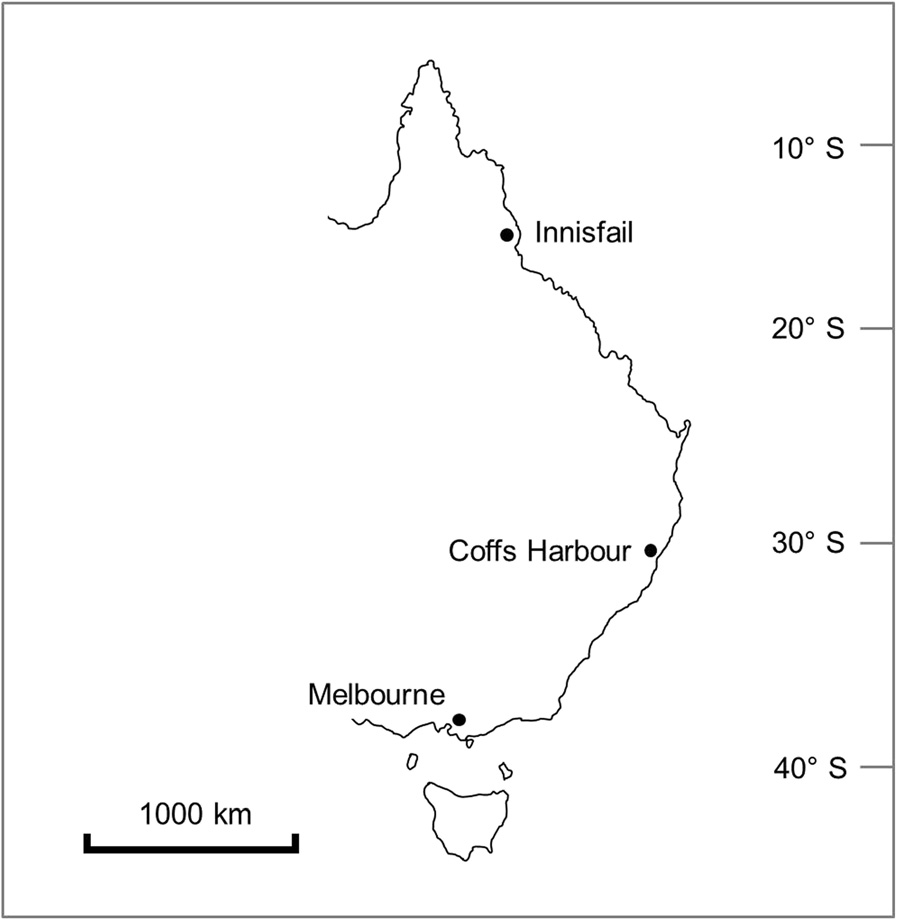
Map of the eastern coast of Australia showing the sampling sites.

If *In*(*2L*)*t* is locally adapted along eastern Australia, we would expect to see differences in the allelic content of inverted chromosomes between sites from different latitudes. Due to the interaction between recombination processes (gene conversion and double crossovers) and selection, we would also expect to see a mosaic of more and less differentiated regions between standard and inverted chromosomes [23] that differ among sites, as well as different patterns of LD within *In*(*2L*)*t* among sites. We test these predictions by examining microsatellite variation within standard and *In*(*2L*)*t* chromosomes at three different sites (Figure 1). Haplotypes were obtained by crossing isofemale lines to an isogenic strain and subtracting the allele present in the isogenic strain from the genotypes of the F_1_ progeny. Under the assumption that microsatellites are neutral, no differences between chromosome arrangements or among the same arrangement from different sites are expected by selection on the microsatellite loci themselves. However, hitchhiking effects aided by extensive LD across the inverted regions, as well as mutations accumulating in chromosomes after hitchhiking events, may lead to genetic differences in the allelic content of *In*(*2L*)*t* along the cline.

## Results

### Marker variation

**Figure 2:**
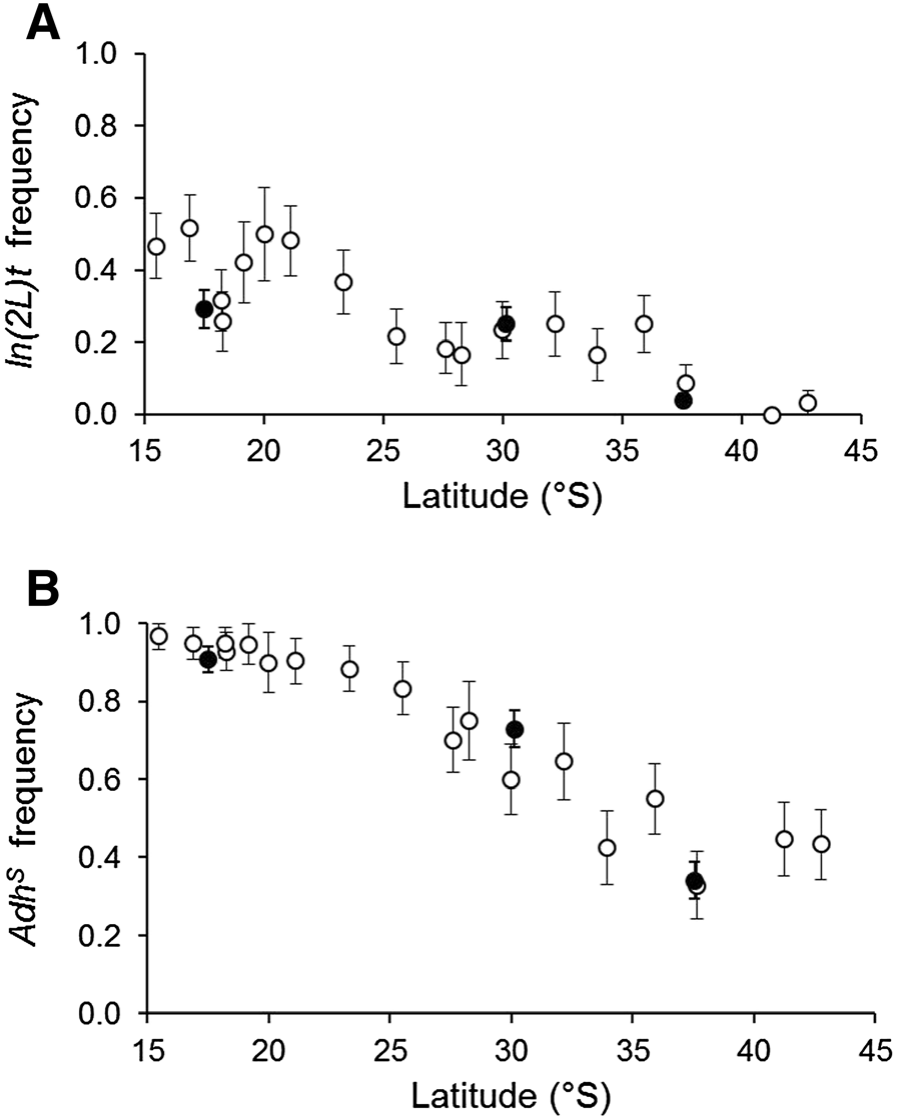
**Relationship between latitude and frequency of *In*(*2L*)*t *(A) and the *Adh***^***S ***^**allele (B) in populations of *Drosophila melanogaster *collected along the east coast of Australia.** Solid circles are data from this study. Open circles are data taken from Umina et al. [28]. Error bars are standard errors.

Large differences in diversity were observed between markers. The number of alleles ranged from two to 23 and gene diversities ranged from 0.01 to 0.83 (Table 1). All individuals from the isogenic strain were homozygous for the same allele at all markers and all F_1_ individuals had a genotype that included at least one allele from the isogenic strain. There were marked differences in the frequencies of the *Adh*^*S*^ allele and *In*(*2L*)*t* among sites. Frequencies of the *Adh*^*S*^ allele ranged from 0.34 to 0.91 and *In*(*2L*)*t* from 0.04 to 0.29. These frequencies were very close to those reported by Umina *et al*. [28], suggesting the crossing scheme did not favour a particular *Adh* allele or chromosome arrangement (Figure 2).

**Table 1:** Details of the markers used in this study

	**Position**			
**Marker**	**Genetic (cM)**	**Cytological**	**No**. **of alleles**	***H***
DROEXPAND	0.7	21C4	7	0.47
DROYANETSB	5	22D1-D2	23	0.80
**AC009392**	7	23A-E	8	0.72
**DS01340**	10	24A1-A2	6	0.50
**AC004373**	12	24F1-F2	9	0.69
**AC004721**	17	25E6	5	0.37
**DROGPDHA**	18	26A1	3	0.51
**AC004758**	18	26A5-B5	14	0.78
**DRONINAC**	27	28A1-A3	4	0.60
**AC004722**	28	28C2-C4	5	0.48
**AC005555**	31	29A1-C1	9	0.77
**AC005889**	35	30A3-A6	7	0.64
**DMBIBGENE**	38	30F	7	0.64
**DMU12269**	39	31A1-A3	21	0.81
**DRODANS**	42	31E	2	0.01
**AC005115**	44	32D2-D4	5	0.43
**G410**	46	33E9-E10	18	0.83
AC006302	48	34C4-D2	10	0.67
AC004118	50	35B2-B3	20	0.83
*Adh*	50	35B3	2	0.35
DRODORSAL	53	36C	10	0.56

Markers in bold type are located within the breakpoints of *In*(*2L*)*t*. *H* is gene diversity.

**Figure 3:**
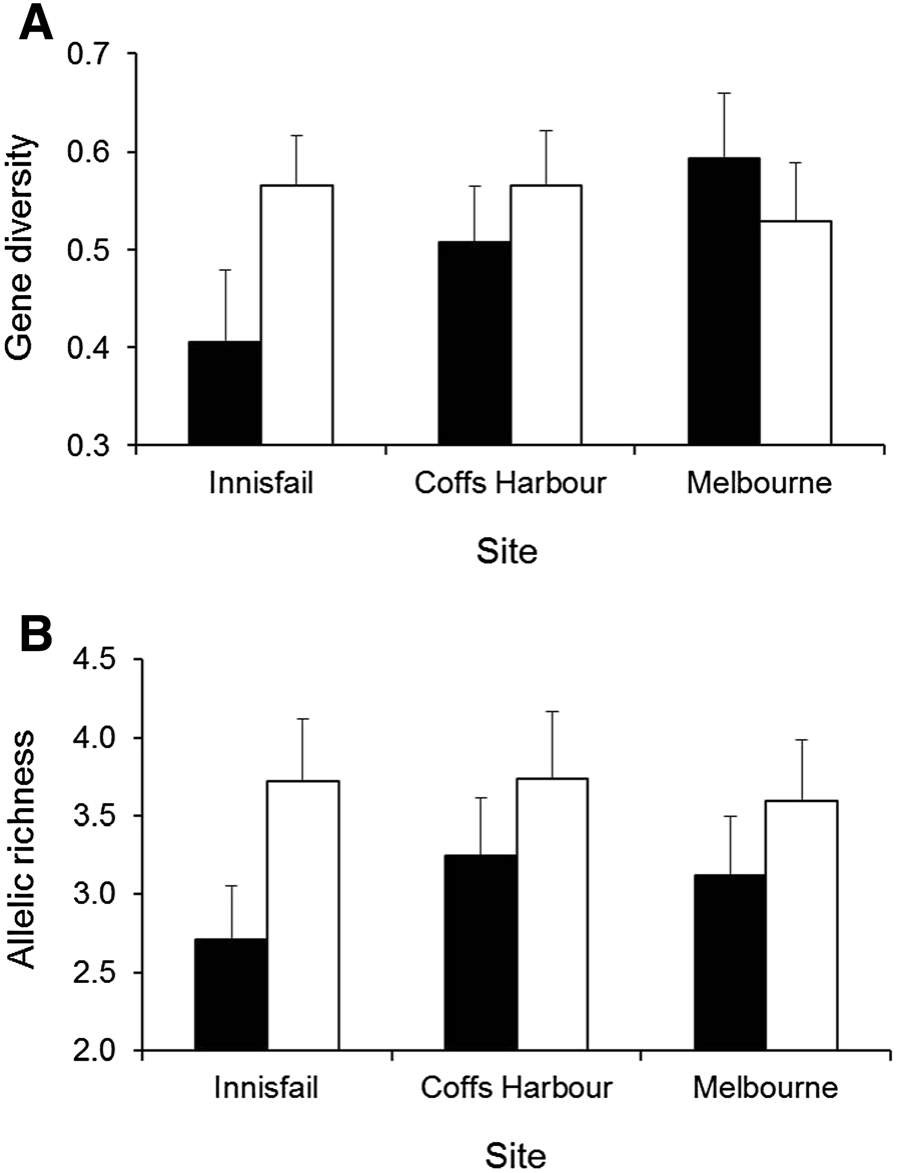
**Levels of genetic variation within *In*(*2L*)*t *(black) and standard arrangement (open) chromosomes.** Gene diversity (**A**) and allelic richness (**B**). Error bars are standard errors.

Genetic diversity tended to be lower in chromosomes with *In*(*2L*)*t* compared to those with the standard arrangement (Figure 3). Pairwise tests between chromosome arrangements at each site revealed significantly higher allelic richness in the standard chromosomes at Innisfail (*P* = 0.014) and when all sites were pooled (*P* = 0.044), but there were no significant differences in allelic richness between chromosome arrangements at Coffs Harbour (*P* = 0.228) or Melbourne (*P* = 0.389). There were also no significant differences between sites within each chromosome arrangement (χ^2^ = 4.30, *P* = 0.116 and χ^2^ = 2.14, *P* = 0.344 for the inverted and standard chromosomes respectively). Gene diversity did not differ significantly between chromosome arrangements at any site (Innisfail: *P* = 0.117; Coffs Harbour: *P* = 0.410; Melbourne: *P* = 0.188) or between sites within standard chromosomes (χ^2^ = 3.76, *P* = 0.152), but there were significant differences between sites in the inverted chromosomes (χ^2^ = 7.36, *P* = 0.025). Pairwise tests indicated that the differences in gene diversity were between the Innisfail and Melbourne sites (*P* = 0.002).

There was a significant heterozygosity excess at the Melbourne site in the inverted chromosomes (Wilcoxon test, *P* = 0.002), indicating a recent severe reduction in effective population size. None of the other site/ chromosome arrangement combinations had higher than expected heterozygosities (Wilcoxon *P*-values ranged from 0.830 to 0.997).

### Genetic differentiation within and between chromosome arrangements

**Figure 4:**
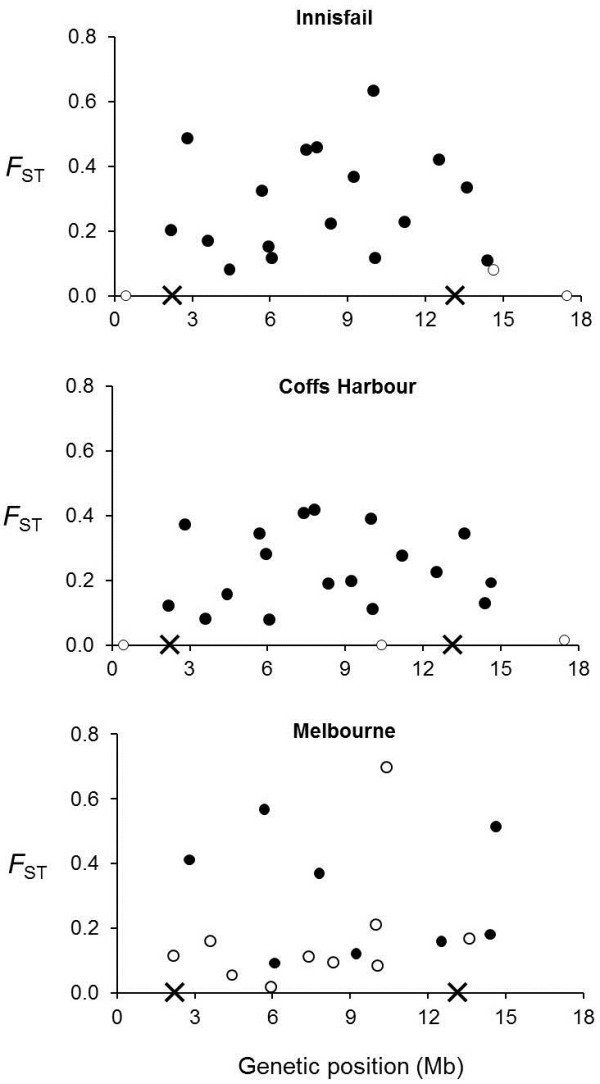
**Marker divergences between chromosome arrangements at each site.** Solid symbols represent markers with significant divergence after correction for multiple comparisons. Open symbols are not significantly different to zero. The crosses on the x-axis depict the locations of the *In*(*2L*)*t* breakpoints.

Nearly all markers within *In*(*2L*)*t* showed significant differentiation between the standard and inverted chromosomal arrangements at the Innisfail and Coffs Harbour sites (Figure 4). Significantly differentiated markers were also evident outside the inversion breakpoints (Figure 4), but they were less common and the divergences were lower (inside mean *F*_ST_ = 0.28 and 0.22, outside mean *F*_ST_ = 0.12 and 0.13). Fewer markers were significantly differentiated at the Melbourne site, a likely consequence of the relatively small number of inverted chromosomes sampled at this site. Overall, patterns of differentiation between inverted and standard chromosomes were quite similar across sites (Spearman’s rank correlations between *F*_ST_ values at different sites were significant, *P* < 0.05 in all cases), but there were notable differences. For example, the region of highest differentiation between chromosome arrangements at Innisfail was in the middle of *In*(*2L*)*t*, but it was more evenly spread across the inversion at Coffs Harbour (Figure 4).

**Figure 5:**
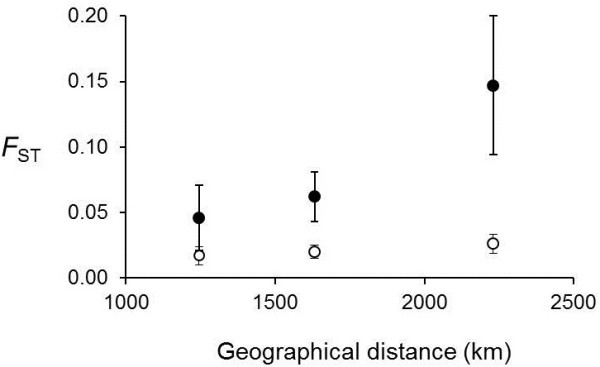
**Relationship between genetic and geographical divergence.** Solid circles are *In*(*2L*)*t* chromosomes. Open circles are standard arrangement chromosomes. Error bars are 95% confidence limits calculated by bootstrapping over loci.

In addition to differentiation between chromosome arrangements at each site, there were significant differences among sites within each chromosome arrangement (inversion: *F*_ST_ = 0.070, *P* < 0.001; standard *F*_ST_ = 0.021, *P* < 0.001). However, the differences among sites within chromosome arrangements were small compared to the differences between inverted and standard chromosomes. AMOVA revealed that 26.9% of the total genetic variation was among chromosome arrangements, while only 2.1% occurred among sites within chromosome arrangements. Levels of differentiation between pairs of sites within each chromosome arrangement increased as the geographical distance between them increased (Figure 5). It was also apparent from non-overlapping 95% confidence limits (CLs) that genetic differentiation between sites was significantly higher within the inverted chromosomes than it was within standard chromosomes (Figure 5).

The outlier analyses identified a single marker, *Adh*, with an excessively high *F*_ST_ value compared to neutral expectations. It was an outlier in the comparison between Innisfail and Melbourne (*P* = 0.002). *Adh* also had a high *F*_ST_ value relative to other markers in the comparison between Coffs Harbour and Melbourne, but was non-significant after correcting for multiple comparisons using a false discovery rate of 10% (*P* = 0.023).

### Linkage disequilibrium and genetic mixing

**Figure 6:**
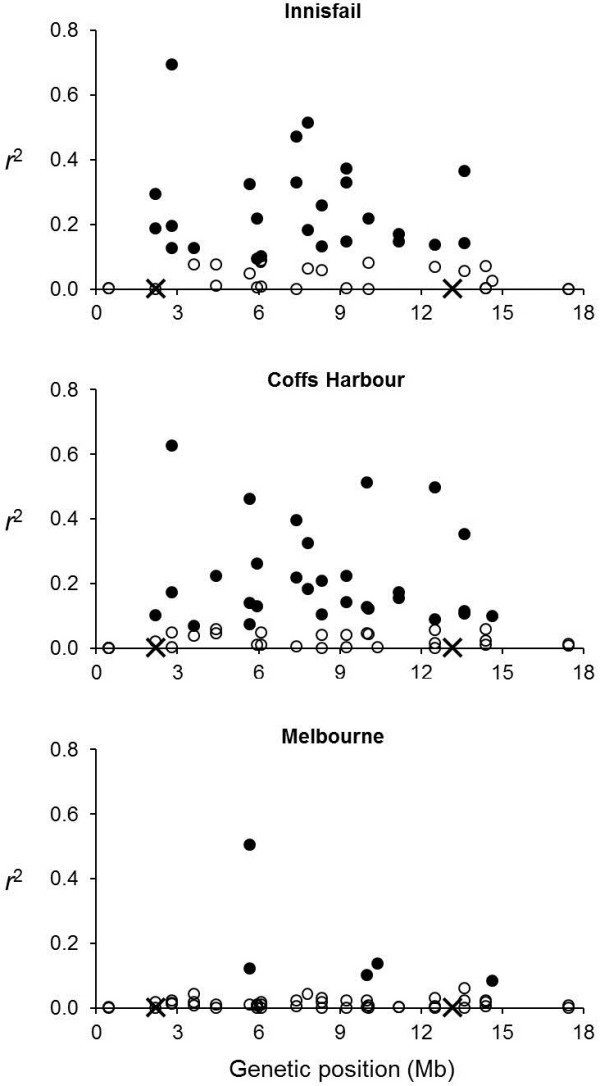
**Linkage disequilibrium between marker alleles and *In*(*2L*)*t*.** Solid circles are markers in significant LD with *In*(*2L*)*t*. Open symbols are not significantly associated with *In*(*2L*)*t*. The crosses on the x-axis depict the locations of the *In*(*2L*)*t* breakpoints.

Associations between marker alleles and *In*(*2L*)*t* were evident at all sites (Figure 6), though they tended to be higher in the low and middle latitude sites (mean *r*^2^ = 0.16 and 0.12 for Innisfail and Coffs Harbour respectively) than in the high latitude site at Melbourne (mean *r*^2^ = 0.03). Nevertheless, associations between alleles and *In*(*2L*)*t* were consistent across sites. Spearman’s rank correlations between *r*^2^ values at different sites were highly significant (*P* < 0.002 in all cases) with *r*_S_ values ranging from 0.48 to 0.84.

**Figure 7:**
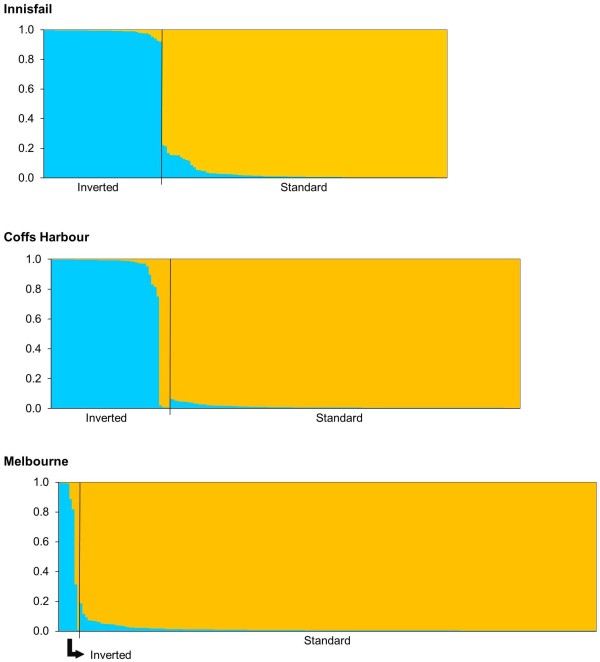
**Summary of the clustering analysis assuming two admixed populations (k = 2).** Each individual is represented by a bar showing the individual’s estimated membership to a particular cluster. Black lines separate samples with different chromosome arrangements.

Cluster analysis on the genetic markers situated within *In*(*2L*)*t* revealed a strong correspondence between membership to a particular cluster and chromosome arrangement (Figure 7). In most cases, individual chromosomes were assigned completely to one cluster, with each chromosome arrangement being represented by a different cluster. However, there were exceptions. Some chromosomes were assigned to the cluster that represented the alternate chromosome arrangement. There were also several chromosomes with a significant proportion of membership to both clusters (Figure 7).

### Decay of linkage disequilibrium and genetic differentiation between chromosome arrangements

**Figure 8:**
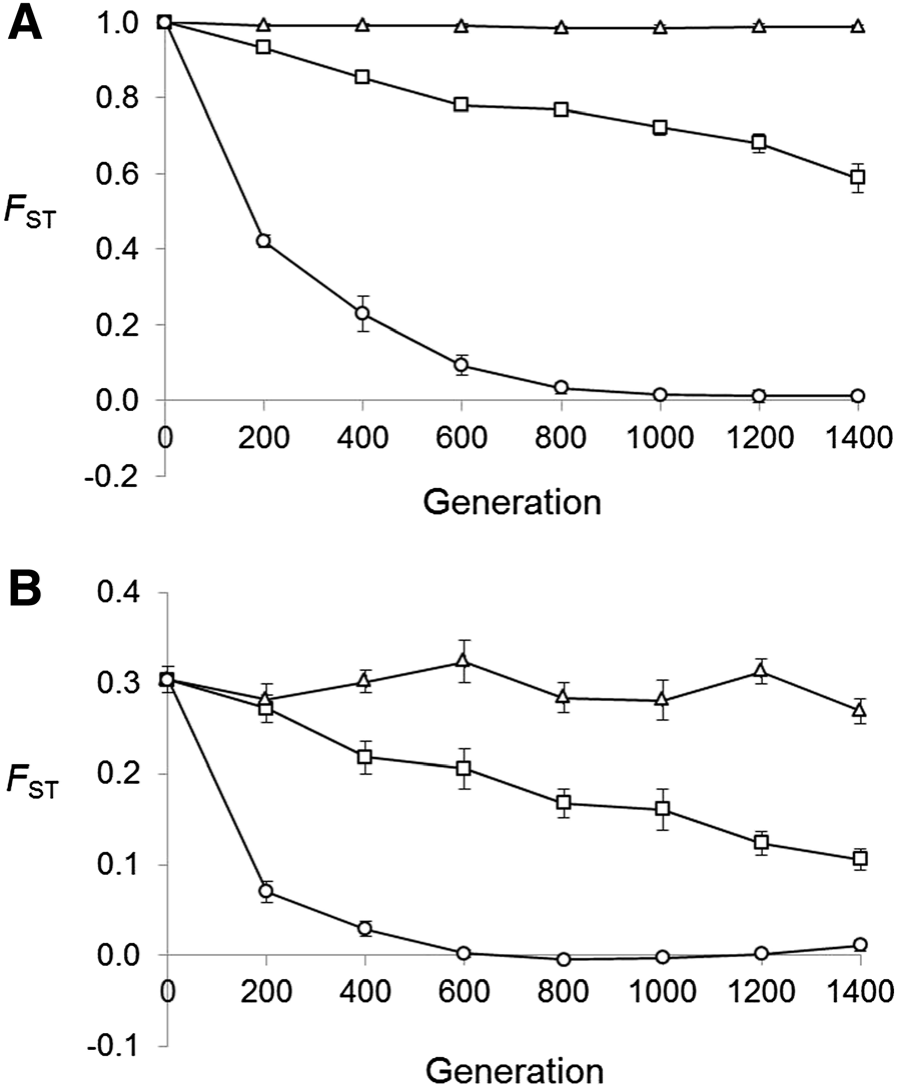
**Mean marker divergences between chromosome arrangements for simulated data generated using different levels of gene exchange.** Rates of gene exchange are zero (triangles), 0.0001 (squares) and 0.00094 (circles) per generation. Simulations were started using maximum (**A**) or moderate (**B**) levels of genetic divergence between chromosome arrangements. Error bars are standard errors calculated by jackknifing over loci.

Our simulations suggest that double-crossovers will homogenize allele frequencies and breakdown LD at loci in the middle of the inversion quickly provided the rate of gene exchange is high. When simulations were run using a rate of gene exchange calculated from map distances (0.0094 per generation), genetic differentiation between chromosome arrangements (subpopulations) declined sharply with *F*_ST_’s close to zero by generation 800, even when the initial allele frequency differences were at a maximum (Figure 8). Declines in genetic divergence were also evident when the rate of gene exchange was set at 0.0001 per generation, but *F*_ST_’s were still well above zero after 1400 generations (Figure 8). Similar patterns were observed when levels of gene diversity and allelic richness were compared between chromosome arrangements. When the level of gene exchange was set at the higher rate, differences in genetic diversity decreased rapidly to be close to zero by generation 800, irrespective of the starting divergence between chromosome arrangements. However, under the lower rate of gene exchange, significant differences in gene diversity and allelic richness (with higher levels of variation in the standard arrangement) were still present at generation 1400. When simulations were started using maximum divergence between chromosome arrangements (i.e. each chromosome arrangement was fixed for a different allele), differences were either non-significant or there was higher genetic diversity in the inverted chromosomes.

No significant LD between marker alleles and the inversion was found in the simulated data sets at generation 1400 when the higher rate of gene exchange was used (mean *r*^2^ = 0.02, much lower than the observed values in Figure 6). However, some LD was detected when the lower rate of gene exchange and maximum levels of genetic divergence between chromosome arrangements was used, with 82.6% (mean *r*^2^ = 0.36) of alleles showing a significant association with the inversion for simulations started at maximum genetic divergence between chromosome arrangements. This dropped to 7.5% (mean *r*^2^ = 0.05) when there was a moderate level of divergence. Therefore the simulations suggest that we observed more LD than expected in all but the most extreme case. Moreover, these estimates assume an effective population size of 10^6^, likely to be an underestimate for *D*. *melanogaster* populations [29].

## Discussion

According to the models proposed by Dobzhansky [3] and Kirkpatrick and Barton [14], the selective value of inversions comes from their ability to hold together sets of locally adapted alleles. However they differ on whether epistatic interactions are necessary among the alleles, and also on whether there are interactions among alleles in inverted and non-inverted chromosomes. Moreover, while the Kirkpatrick and Barton [14] model focuses on the spread of an inversion in populations due to the combination of favourable alleles, Dobzhansky’s verbal arguments focused on combinations of alleles that worked together within as well as between populations, and emphasized that different combinations might be favoured in different populations even when they were in the same chromosomal rearrangement.

Both these models predict that the genetic content of inverted and standard arrangements should differ, and that differences may also develop within arrangements at different sites. Consistent with this, our data show there is strong differentiation between chromosome arrangements at all sites, particularly for some chromosomal regions. They also show significant differentiation among *In*(*2L*)*t* chromosomes sampled from different sites along a latitudinal cline that is significantly greater than levels of differentiation observed at the same markers in standard chromosomes. While a small number of allozyme studies have shown differences in allelic content of the same inversion among populations in *Drosophila* (e.g., [20,21,30]), they are characterized by low sample sizes and analyses involving only a few loci [13]. Due to the low frequency of *In*(*2L*)*t* at high latitudes, our high latitude site also had a low sample size. However, this is not the case for the low and mid latitude sites (*n* ≥ 44 chromosomes), which also show significant differentiation between chromosome arrangements and between sites within the same chromosome arrangement.

A similar pattern of differentiation between chromosome arrangements that we have observed here has been found in other studies [23,25], whose authors have argued that such patterns arise through directional selection maintaining divergence between chromosome arrangements at specific loci in the face of genetic exchange between them. In our case, where there has been a recent introduction, is a low incidence of recombination adequate in explaining these patterns, without the need to invoke selection? The clustering analysis indicates that some inverted chromosomes had the allelic content of standard chromosomes, suggesting an ongoing process of recombination between the arrangements. It is interesting to note that these chromosomes were absent in the low latitude site, suggesting stronger selection against standard chromosome alleles in the inverted background at low latitudes.

Recombination is also suggested by the absence of strong genetic differentiation between northern and southern Australian populations in the regions spanned by *In*(*2L*)*t*, even though there is a cline for this inversion [31]. Recombination by crossing over should be more effective away from inversion breakpoints that maintain a strong historical signature, which may include *In*(*2L*)*t* in *Drosophila*[32]. However, in our analysis there is no tendency for genetic differentiation to decrease towards the centre of this large inversion. Instead a combination of LD and selection across multiple loci along the inversion may explain this pattern, although the simulations suggest that we cannot entirely rule out initial colonization of Australia by individuals with inverted and standard arrangements fixed for different alleles and/or a lower than expected rate of gene exchange.

Differences in genetic variation between chromosome arrangements may reflect the fact that the inversion is derived from the standard arrangement, although it is not likely that this event, estimated to be some time ago (~ 160,000 years, [32]), would have much ongoing effect on microsatellite variation. Alternatively, the differences may reflect a lower number of founders with the inverted arrangement at the time of colonization (more than 100 years ago, [33]). However, again, we would expect this difference to break down due to recombination and gene conversion unless the effective size of a population is particularly low. Indeed our simulations show that differences in allelic richness and gene diversity between chromosome arrangements would disappear relatively quickly, within the time since colonization, if the frequency of double crossovers in heterokaryotypes matches the level expected from map distances. The extent of LD between markers and the inversion was also much lower in the simulated data than was observed, even when the level of gene exchange between arrangements was low, except when the starting divergences between arrangements were extreme.

The outlier analysis provided evidence of directional selection on the *Adh* polymorphism along the cline, but only in standard chromosomes. This result is not unexpected given the strong association between the *Adh*^*F*^ allele and standard chromosomes. Based on data from previous studies and their own, Veuille *et al*. [34] reported only two inverted chromosomes with an *Adh*^*F*^ allele in a list of 1002 chromosomes, although 49 were expected at random. In our survey of 538 chromosomes, including 97 inversions (18.0%) and 205 *Adh*^*F*^ alleles (37.7%), we found only two *In*(*2L*)*t* chromosomes with an *Adh*^*F*^ allele. The proportion of *In*(*2L*)*t* and *Adh*^*F*^ chromosomes in our study was slightly higher, but not significantly different (Yates Chi-square = 0.01, *P* = 0.914), to the proportion reported by Veuille *et al*. [34]. The strong association between standard chromosomes and *Adh*^*F*^ has been attributed to both selection and historical processes. Based on molecular variation at *Adh*, Veuille *et al*. [34] suggests that the historical explanation is more likely, with the lack of *In*(*2L*)*t* and *Adh*^*F*^ chromosomes due to recent contact between different haplotypes that evolved in isolation.

## Conclusions

Genetic markers situated within the inverted regions showed high levels of differentiation between chromosome arrangements, despite the potential for recombination between chromosome arrangements as emphasized by the clustering analysis. This strong differentiation particularly away from inversion breakpoints suggests that patterns of variation are partly influenced by selection and LD. Simulations provide support for this view, but more accurate estimates of gene exchange between chromosome arrangements are required and we cannot entirely rule out strong differentiation of markers in the initial colonization of Australia. There was also some evidence for the development of population differentiation within arrangements and particularly the inverted arrangement. Levels of genetic variation were lower within the inverted arrangement, which may reflect founder events, but selection facilitated by LD is also suggested because there was no evidence of severe reductions in effective population size. Additional research on combinations of chromosomal regions should help indicate whether there are epistatic interactions among regions.

## Methods

### Fly collections

Wild *D*. *melanogaster* were collected from three locations on the east coast of Australia between January and June in 2008 (Figure 1). These sites represent ends of the latitudinal clines in *In*(*2L*)*t* and the *Adh* F/S polymorphism documented in Knibb *et al*. [35] and Oakeshott *et al*.[27]. They included a low latitude site at Innisfail (17.51°S 146 00°E), a high latitude site at Melbourne (37.56°S 145 10°E) and a mid-latitude site at Coffs Harbour (30.14°S 153 49°E). The geographical distance between these sites ranged from 1244 to 2230 km.

From each site between 118 and 189 isofemale lines were established from individual field collected females. These lines were maintained at 25°C under continuous light on a sugar (1.6% w/v), agar (3.2%), yeast (3.2%) and potato (1.6%) medium that was always treated with an antifungal agent (0.14% nipagin) and antibiotics (2% dihydrostreptomycin and 0.6% penicillin added to the medium surface). After two to 12 generations in the laboratory, virgin females from each isofemale line were crossed to males from the isogenic *y*[*1*]; *cn*[*1*] *bw*[*1*] *sp*[*1*] strain (FlyBase ID: FBst0002057). The progeny from these crosses were preserved in 100% ethanol and stored at -20°C for genotyping.

### Genotyping

DNA extraction from individual flies, PCR protocols, and allele scoring followed methods outlined in Gockel *et al*. [36]. Each fly was genotyped at 20 microsatellite loci and molecular markers for the *Adh* and *In*(*2L*)*t* polymorphisms. All molecular markers were located on the left arm of chromosome 2, close to or within the breakpoints of *In*(*2L*)*t* (Table 1). Primer sequences and information about microsatellite loci are provided in Additional file 1. Primer sequences and protocols for genotyping the *Adh* and *In*(*2L*)*t* polymorphisms are described in Umina *et al*. [28] and Andolfatto *et al*. [32] respectively. A maximum of three F_1_ progeny (mean = 1.2) were genotyped from each isofemale line, with a total of 154 flies from the Innisfail site, 179 flies from the Coffs Harbour site and 205 flies from the Melbourne site. Eight individual flies from the isogenic *y*[*1*]; *cn*[*1*] *bw*[*1*] *sp*[*1*] strain were also genotyped for each of the molecular markers.

### Data analysis

A haploid, multilocus data set of known gametic phase was created by subtracting the allele present in the isogenic *y*[*1*]; *cn*[*1*] *bw*[*1*] *sp*[*1*] strain (the sire) from the genotype of each F_1_ individual at each genetic marker. The chromosome arrangement for each haplotype was also determined in the same way using the genotype of the *In*(*2L*)*t* genetic marker. These data were used for all subsequent analyses.

For each site/chromosome arrangement combination, the level of genetic variation was quantified by calculating allelic richness (a measure of the number of alleles independent of sample size) and gene diversity using the fstat software package [37]. Differences in genetic variation between chromosome arrangements at each site were tested using a Wilcoxon’s signed-rank test (for paired comparisons between two groups) and differences among sites within each chromosome arrangement were tested using a Friedman’s ANOVA (for multiple paired comparisons). Genetic differentiation between chromosome arrangements and among sites within each chromosomal arrangement was assessed by calculating Weir & Cockerham’s [38] estimator of *F*_ST_. Pairwise *F*_ST_ values, 95% confidence limits for these values and tests for differentiation among sites were calculated with the fstat software package [37]. We used analysis of molecular variance (AMOVA) to partition genetic variation between chromosome arrangements and among sites within chromosome arrangements. Linkage disequilibrium between marker alleles and *In*(*2L*)*t* were quantified using the *r*^2^ coefficient. Estimates of *r*^2^, the significance level of the disequilibrium and AMOVA were calculated with the arlequin version 3 software package [39].

Tests for selection acting on marker loci were carried out using the *F*_ST_ outlier approach [40,41] and were performed with the lositan software package [42]. The method involves evaluating the relationship between *F*_ST_ and expected heterozygosity in an island model of migration with neutral markers. This distribution is used to identify excessively high or low *F*_ST_ values compared to neutral expectations. Such outlier loci are candidates for being subject to selection. Simulations were run using 10 000 replications, 95% confidence intervals and the neutral and forced mean options. An infinite allele mutation model was assumed. Analyses using the stepwise mutation model were also carried out, but they provided similar results, so only those with the infinite allele mutation model are presented.

To assess the extent of genetic mixing between chromosome arrangements at each site, a cluster analysis was performed using the program structure, version 2.1 [43]. We assumed the presence of two genetic clusters (k = 2) to represent each of the chromosome arrangements segregating in the populations. Chromosomes were assigned a membership coefficient, which is the proportion of the genome that is derived from a particular cluster. structure was run with the admixture model and correlated allele frequencies [43,44]. Five independent runs were performed using 100000 iterations, with a burn-in period of 10000 iterations.

Finally, tests for a severe reduction in effective population size (population bottleneck) were performed for each site/chromosome arrangement combination using the software package bottleneck[45]. The method used was based on the principle that that the number of alleles decreases faster than expected heterozygosity after a bottleneck [46]. In this situation, expected heterozygosity should be higher than the equilibrium heterozygosity predicted in a stable population from the observed number of alleles. Following the authors’ recommendation for microsatellite data, we used a two-phase model (TPM) with 95% single-step mutation and 5% multiple-step mutations (and a variance among multiple steps of 12). A Wilcoxon signed rank test was run to determine whether each sample had a significant excess of heterozygosity.

With the exception of the LD calculations and tests for loci under selection, analyses were performed using markers situated within the breakpoints of *In*(*2L*)*t* only. Because sampling multiple chromosomes from within isofemale lines may influence LD, only a single chromosome from each isofemale line were used for the LD analysis. Corrections for multiple comparisons were applied to all tests.

### Decay of linkage disequilibrium and genetic differentiation between chromosome arrangements

To determine whether there has been sufficient time for gene exchange to breakdown LD and genetic differentiation between chromosome arrangements in the middle part of *In*(*2L*)*t* in Australian populations, simulated data sets were created using a modified version of the EASYPOP 2.0.1 computer program [47]. The simulations were based on a simple model consisting of two haploid subpopulations, representing each of the alternate chromosome arrangements. These two subpopulations were allowed to exchange genes between each other at a rate equivalent to the expected frequency of double crossovers and gene conversions occurring within *In*(*2L*)*t* each generation. Unfortunately there are no estimates of these processes for microsatellite markers, and two rates of gene exchange were used. The first was a rate of 9.4 × 10^–4^ per generation, which is equal to the probability of a crossover event occurring within a 5 cM region (1.2 – 3.4 Mb) inside each of the inversion breakpoints in heterokaryotypes (0.05 × 0.05 × 2*q*(1 – *q*), where *q* is the frequency of *In*(*2L*)*t*). The second was a rate of 1.0 × 10^–4^ per generation, which is an estimate of the rate of double crossovers between segregating inversions in *Drosophila* based on phenotypic markers located near the centre and inversion breakpoints [48,49].

The total number of chromosomes (i.e. both subpopulations combined) was set at 2.0 × 10^6^ (*N*_e_ ~ 10^6^, [50]). However, the number of chromosomes in each subpopulation varied to reflect the different frequencies of standard and *In*(*2L*)*t* chromosomes segregating in tropical populations (0.75 and 0.25 respectively). The mutation rate was set at 5.65 × 10^–6^, which is the weighted average of microsatellite mutation rates observed in *D*. *melanogaster*[51,52]. Both a single step and two-phased models of mutation were used. However, because both mutation models gave qualitatively similar results, only the results with the single step mutation model are presented.

The simulations ran for 1400 generations (the estimated number of generations *D*. *melanogaster* has been in Australia assuming 10 generations per year [33]) and were based on 10 loci with free recombination and a maximum of eight alleles per locus (the average number of alleles at each locus). Simulations were started with either maximum or intermediate (*F*_ST_ ~ 0.3) allele frequency differences between subpopulations. In models with maximum genetic divergence, subpopulations were fixed for different alleles at each locus. Subpopulations with intermediate genetic divergences were set up by randomly selecting alleles from all possible allelic states for one subpopulation and randomly selecting alleles from a subset of possible allelic states for the other subpopulation. This also resulted in a difference in allelic richness between subpopulations, which is likely in natural populations given the smaller *N*_e_ and more recent origin of inverted chromosomes.

Each generation 44 chromosomes (the minimum sample size from mid and low latitude sites in our study) were sampled from each subpopulation and used to calculate the level of divergence between the subpopulations. Simulated data sets from generation 1400 were also analyzed with arlequin to assess levels of LD.

## Competing interests

The authors declare that they have no competing interests.

## Authors’ contributions

WJK and AAH conceived and designed the study. WJK collected and analysed the data. Both authors wrote the manuscript. Both authors read and approved the final manuscript.

## Supplementary Material

Additional file 1: Table S1 Repeat type and primer sequences for microsatellite loci. Click here for file
