# Supplementary material for: Patterns of genetic variation across inversions: geographic variation in the In(2L)t inversion in populations of Drosophila melanogaster from eastern Australia
Source: BMC Evol Biol. 2013 May 20;13:100. doi: 10.1186/1471-2148-13-100 (PMC3667013; doi:10.1186/1471-2148-13-100)
Supplement: Additional file 1: Table S1 — Repeat type and primer sequences for microsatellite loci. [file 1471-2148-13-100-S1.docx]

**Table S1:** Repeat type and primer sequences for microsatellite loci

| Locus name | Repeat type | Forward primer sequence | Reverse primer sequence | Reference |
| --- | --- | --- | --- | --- |
| DROEXPAND | (CAG)8 | gtgatcgatcccgctgtc | tccggtttccaattagcttg | [[1](#_ENREF_1)] |
| DROYANETSB | (GT)20 | taatggggaatgggtgaatg | gccgtgctctttctcttacg | [[1](#_ENREF_1)] |
| **AC009392** | (GA)9 | tgcgcagtaaagagagcaga | ggcgaatcgcgtagaaaat | [[2](#_ENREF_2)] |
| **DS01340** | (AG)9 | ggagcgcaatgctgtttaagt | ggagtagtgcctgtctcggac | [[3](#_ENREF_3)] |
| **AC004373** | (AT)15 | aatgcgtgtgtttggatgaa | gtcccagtctcccagtgaaa | [[4](#_ENREF_4)] |
| **AC004721** | (CA)8 | gcctgcgggaatacttttt | ttgctgggtccctttttatg | [[5](#_ENREF_5)] |
| **DROGPDHA** | (CT)7 | cattggaaaagtgagcggat | ttggtttgcactccacacat | [[4](#_ENREF_4)] |
| **AC004758** | (TG)11 | tgctttcgctttcggtatct | aacggagtgcctatgcatt | [[4](#_ENREF_4)] |
| **DRONINAC** | (AT)10 | tttgtcaatctctcacagcagg | gcccgagtacatttattcaagc | [[6](#_ENREF_6)] |
| **AC004722** | (GA)8 | gcagatgcttcagctccatt | ctttgctctgcaaggatgtg | [[7](#_ENREF_7)] |
| **AC005555** | (TG)12 | ggttgctgggagaaagac | gccacacattcgcatctc | [[4](#_ENREF_4)] |
| **AC005889** | (CA)13 | gcgtggctggcatatagagt | taagccccctcgtgtaattg | [[7](#_ENREF_7)] |
| **DMBIBGENE** | (CAG)5 | tcgcaaggatcagcggtgac | ttgggcctcagcggcagcat | [[modified from 8](#_ENREF_8)] |
| **DMU12269** | (AAC)7 | tgggatccgtggatcatagt | attcgggaatgaggacactg | [[5](#_ENREF_5)] |
| **DRODANS** | (CAG)5 | tgcccagcatcacatgatac | ggtttttatggaagagaggg | [[4](#_ENREF_4)] |
| **AC005115** | (GT)7 | cgaagtggatccatcgaaat | caccctggactacctgcaat | [[2](#_ENREF_2)] |
| **G410** | (CT)11 | ttcggctctttgtttgcttg | aagcttaaaccgatcgaaaac | [[9](#_ENREF_9)] |
| AC006302 | (AAT)11 | tgttttccatgccagctagt | gcccggaaaattcttgttta | [[4](#_ENREF_4)] |
| AC004118 | (CT)15 | ccaacttgggcgagagaatt | gcttaattgcctcactgtgc | [[4](#_ENREF_4)] |
| DRODORSAL | (CAG)9 | ctgatgtcgcccaaccatcc | ccgccaggattgccaaaggg | [[8](#_ENREF_8)] |

Loci in bold type are located within the breakpoints of *In(2L)t*.

**References**

1. Goldstein DB, Clark AG: **Microsatellite variation in North American populations of *Drosophila melanogaster***. *Nucleic Acids Res* 1995, **23**:3882-3886.

2. Gockel J, Robinson SJW, Kennington WJ, Goldstein DB, Partridge L: **Quantitative genetic analysis of natural variation in body size in *Drosophila melanogaster***. *Heredity* 2002, **89**:145-153.

3. Schlötterer C: unpublished.

4. Colson I, Macdonald SJ, Goldstein DB: **Microsatellite markersfor interspecific mapping of *Drosophila simulans* and *D. sechellia***. *Mol Ecol* 1999, **8**:1951-1955.

5. Colson I: unpublished.

6. Schug MD, Wetterstrand KA, Gaudette MS, Lim RH, Hutter CM, Aquadro CF: **The distribution and frequencies of microsatellite loci in *Drosophila melanogaster***. *Mol Ecol* 1998, **7**:57-70.

7. Gockel J: unpublished.

8. Michalakis Y, Veuille M: **Length variation of CAG/CAA trinucleotide repeats in natural populations of *Drosophila melanogaster* and its relation to the recombination rate**. *Genetics* 1996, **143**:1713-1725.

9. Harr B, Zanger B, Brem G, Schlötterer C: **Conservation of locus-specific microsatellite variability across species: a comparison of two *Drosophila* sibling species, *D. melanogaster* and *D. simulans***. *Mol Biol Evol* 1998, **15**:176-184.
